# The Gly82Ser polymorphism in the receptor for advanced glycation endproducts increases the risk for coronary events in the general population

**DOI:** 10.1038/s41598-024-62385-5

**Published:** 2024-05-21

**Authors:** Helena Grauen Larsen, Jiangming Sun, Marketa Sjögren, Yan Borné, Gunnar Engström, Peter Nilsson, Marju Orho-Melander, Isabel Goncalves, Jan Nilsson, Olle Melander, Alexandru Schiopu

**Affiliations:** 1 Department of Clinical Sciences Malmö, Lund University, 21428 Malmö, Sweden; 2 Department of Cardiology, Skåne University Hospital Malmö, 21428 Malmö, Sweden; 3 Department of Internal Medicine, Skane University Hospital Lund, 22242 Lund, Sweden; 4 Department of Translational Medicine, Lund University, 21428 Malmö, Sweden; 5 Nicolae Simionescu Institute of Cellular Biology and Pathology, 050568 Bucharest, Romania

**Keywords:** Biomarkers, Predictive markers, Genetics research

## Abstract

The receptor for advanced glycation endproducts (RAGE) has pro-inflammatory and pro-atherogenic effects. Low plasma levels of soluble RAGE (sRAGE), a decoy receptor for RAGE ligands, have been associated with increased risk for major adverse coronary events (MACE) in the general population. We performed a genome-wide association study to identify genetic determinants of plasma sRAGE in 4338 individuals from the cardiovascular arm of the Malmö Diet and Cancer study (MDC-CV). Further, we explored the associations between these genetic variants, incident first-time MACE and mortality in 24,640 unrelated individuals of European ancestry from the MDC cohort. The minor alleles of four single nucleotide polymorphisms (SNPs): rs2070600, rs204993, rs116653040, and rs7306778 were independently associated with lower plasma sRAGE. The minor T (vs. C) allele of rs2070600 was associated with increased risk for MACE [HR 1.13 95% CI (1.02–1.25), *P* = 0.016]. Neither SNP was associated with mortality. This is the largest study to demonstrate a link between a genetic sRAGE determinant and CV risk. Only rs2070600, which enhances RAGE function by inducing a Gly82Ser polymorphism in the ligand-binding domain, was associated with MACE. The lack of associations with incident MACE for the other sRAGE-lowering SNPs suggests that this functional RAGE modification is central for the observed relationship.

## Introduction

Atherosclerotic coronary artery disease (CAD) and its complications are the leading causes of death worldwide^1^. Atherosclerosis is a chronic, low grade inflammation in the artery wall, initiated by lipid accumulation and oxidation^2^. Inflammatory cells and their secreted mediators play major roles in both atherogenesis and in plaque vulnerability and rupture. The multiligand immune receptor for advanced glycation endproducts (RAGE) has been implicated in several aspects of the pathogenesis of the disease. When engaged, RAGE promotes inflammation by triggering cell activation and production of pro-inflammatory cytokines, cell migration and tissue infiltration^3,4^. RAGE is expressed on several cell populations that participate in atherogenesis, including endothelial cells, smooth muscle cells, neutrophils and mononuclear phagocytes, and is extensively upregulated during inflammation^3,4^. Known RAGE ligands are AGE’s (advanced glycation endproducts), S100/calgranulin family proteins and HMGB-1 (high mobility group box protein-1)^3^. Following proteolytic cleavage from the cell surface or alternative splicing, RAGE is released into the circulation in a soluble form (sRAGE)^5,6^. sRAGE has been shown to serve as a decoy receptor for RAGE ligands in humans, interfering with the binding and activation of cell-bound RAGE^7^. In experimental settings, sRAGE has demonstrated anti-inflammatory and atheroprotective properties, and has been proposed as a potential treatment for cardiovascular disease (CVD) and diabetes^8,9^. The potential atheroprotective properties of sRAGE are further supported by our previous findings in a CVD-free subpopulation from the Malmö Diet and Cancer study (MDC). We have shown that systemic sRAGE levels are inversely associated with the progression of intima-media thickness (IMT) in the carotid artery, and with the long-term risk for incident major adverse coronary events (MACE) and total mortality^10^. The relationships between cell-bound RAGE and the presence of sRAGE in plasma are, however, unknown. It remains to be determined whether sRAGE has an active protective role against CVD development in humans, or whether it is only a biomarker reflecting the expression, activation and cleavage of cell-bound RAGE.

In the present study we explored the potential links between genetic determinants of sRAGE levels in plasma and CVD risk in a population-based cohort. In an initial step, we performed a genome-wide association study (GWAS) in 4338 individuals from the cardiovascular arm of the MDC study (MDC-CV) in order to identify genetic determinants of sRAGE. Further, we explored the associations between the sRAGE-associated single nucleotide polymorphisms (SNPs), baseline IMT and IMT progression in the common carotid artery during a median follow-up of 16.5 years. We also analyzed the relationships between sRAGE-associated SNPs and incident first-time MACE and mortality in the entire MDC cohort of 24,640 individuals. The median follow-up time of the cohort was 21.2 years.

## Methods

### Study population

**Figure 1:**
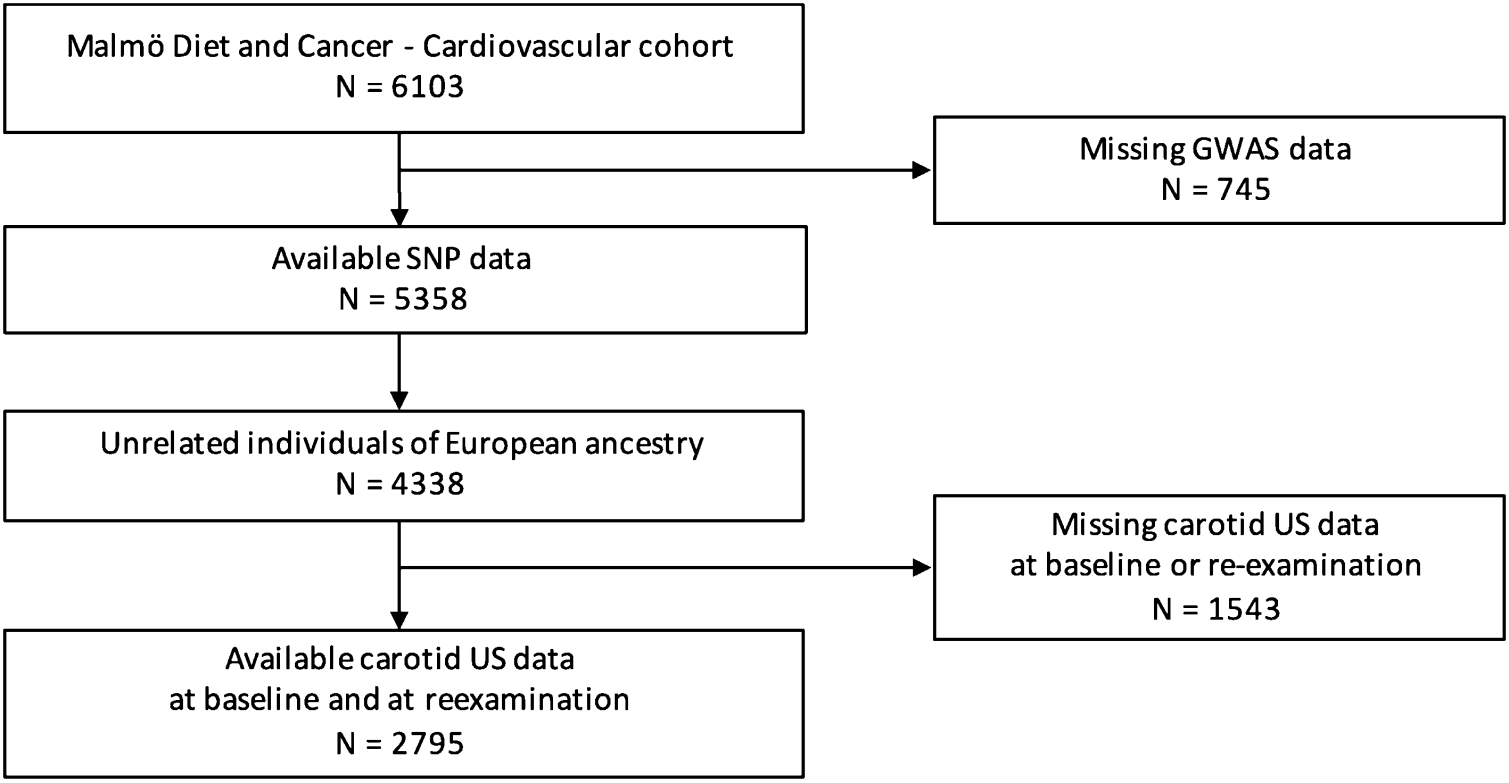
Subject inclusion and follow-up in the MDC-CV cohort. Flow diagram of subject inclusion and exclusion in the cardiovascular arm of the Malmö Diet and Cancer (MDC-CV) study. US, ultrasound; SNP, single nucleotide polymorphism.

**Figure 2:**
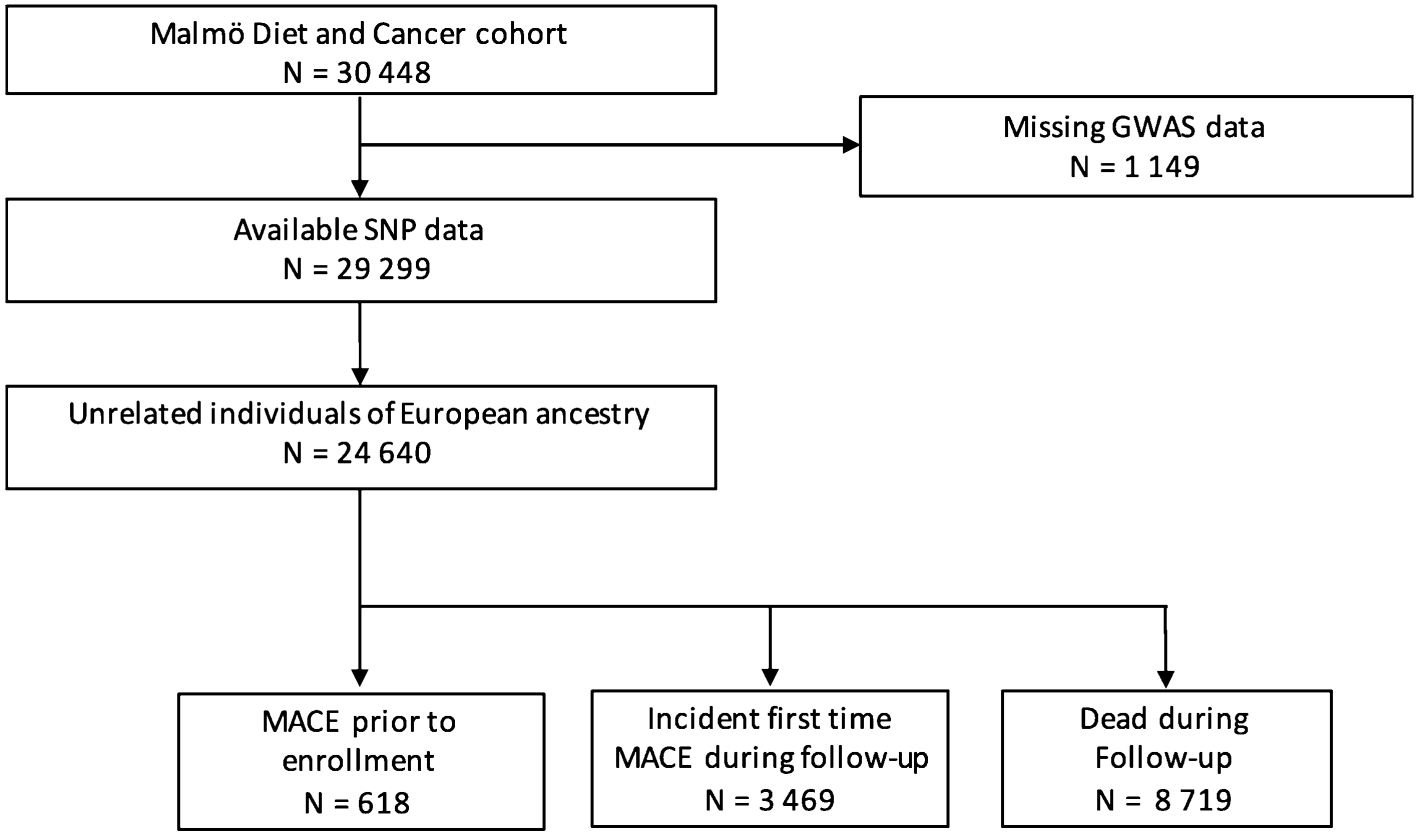
Subject inclusion, follow-up and outcome in the entire MDC cohort. Flow diagram of subject inclusion, exclusion and outcome data in the Malmö Diet and Cancer (MDC) study. US, ultrasound; SNP, single nucleotide polymorphism; MACE, major adverse coronary event.

The MDC cohort of 30,448 subjects and the MDC-CV arm of 6103 individuals were used in this study. The flowcharts describing the inclusion and exclusion criteria are presented in Figs. 1 and 2. The MDC study included a random sample of individuals from Malmö, Sweden, born between 1926 and 1945 and included between March 1991 and September 1996^11^. All participants filled in a self-administered questionnaire and went through a clinical examination and blood sample collection at baseline. Information about smoking habits and current medication was collected from the questionnaire. Diabetes mellitus at baseline was defined as self-reported history of a diabetes diagnosis made by a physician or diabetes medication in the questionnaire or fasting whole blood glucose ≥ 6.0 mmol/L (corresponding to plasma glucose of ≥ 7.0 mmol/L). Between October 1991 and February 1994 a randomly selected subgroup of 6103 MDC participants was included into a study focusing on carotid artery disease epidemiology (MDC-CV)^12^. This subgroup underwent ultrasonographic measurement of IMT in the right common carotid artery at baseline. A plasma aliquot collected at baseline from MDC-CV participants was sent for sRAGE analysis. The characteristics of the cohort are presented in Table 1. The participants were invited to undergo a carotid artery IMT reexamination between 2007 and 2012, completed by 3693 subjects^13^, out of which 2795 had a complete set of genetic data and sRAGE measurements (Fig. 1). The median time from baseline to IMT reexamination was 16.5 (15.4–17.8) years. The entire MDC cohort was used to analyze the association between the identified sRAGE-associated SNPs and clinical outcomes. Genetic and clinical data were available in 29,299 participants (Fig. 2). Of these, 99% were of European descent as estimated by mapping their genotypes to HapMap 3 reference data. A population of 24,640 unrelated individuals of European ancestry with available GWAS data were included in the study (Fig. 2). All participants gave informed consent. The ethics committee at Lund University approved the study, which was conducted in accordance with the Declaration of Helsinki.

**Table 1:** Baseline characteristics of the MDC-CV cohort.

Characteristics	N = 4338
Age (years)	58 (52–63)
Male sex, n (%)	1728 (39.8)
BMI (kg/m^2^)	25.1 (23.0–27.8)
Diabetes, n (%)	324 (7.5)
Current smoking, n (%)	935 (21.6)
Medication use	
Statins, n (%)	68 (1.6)
Blood pressure medication, n (%)	695 (16.0)
Lipids	
LDL (mmol/L)	4.1 (3.5–4.8)
HDL (mmol/L)	1.3 (1.1–1.6)
TG (mmol/L)	1.2 (0.9–1.6)
Blood pressure	
Systolic (mmHg)	140 (128–150)
Diastolic (mmHg)	86 (80–92)
eGFR	73.6 (65.3–82.7)
sRAGE (au)	19.9 (16.2–24.1)
IMT	
Baseline (mm)	0.74 (0.66–0.84)
Follow-up (mm)	0.89 (0.79–1.03)
Increase (mm)	0.16 (0.07–0.26)
Increase yearly (mm)	0.01 (0.004–0.016)

The data are presented as median (interquartile range) for the continuous variables.

MACE, major adverse coronary event; sRAGE, soluble receptor for advanced glycation endproducts; BMI, body mass index; LDL, low density lipoprotein; HDL, high density lipoprotein; TG, triglyceride; eGFR, estimated glomerular filtration rate.

### IMT and carotid ultrasound

The carotid ultrasonographic examination protocol has been described in detail earlier^14^. In short, a B-mode ultrasonography system (Acuson XP/4 for the baseline examination and Acuson Sequoia for the follow-up examination) was used to scan the right common carotid artery in systole, at the top of the R-wave on ECG, by certified sonographers. IMT was measured as the mean wall thickness in a 1 cm-wide window proximal to the bifurcation, according to a standardized protocol. Each image was analysed without knowledge of subject identification code, to minimize observer bias.

### Clinical outcomes

To obtain information on incident and prevalent events we linked the personal identification code of each subject to the National Hospital Discharge register, the National Cause of Death Registry and the Swedish Coronary Angiography and Angioplasty Registry (SCAAR)^14–16^. MACE was defined on the basis of an acute coronary event or a revascularization procedure (coronary by-pass surgery or percutaneous coronary intervention). An acute coronary event was defined as a fatal or non-fatal myocardial infarction on the base of International Classification of Diseases, ninth revision (ICD-9) code 410 and International Classification of Diseases tenth revision (ICD-10) code I21 or death attributable to ischemic heart disease (ICD-9 codes 412 & 414 or ICD-10 codes I22, I23 and I25). Coronary revascularizations were based on the SCAAR procedure codes 3065, 3066, 3068, 3080, 3092, 3105, 3127, or 3158 in the Op6 system or as procedure code FN in the KKÅ97 system. The participants were followed from baseline until the first incident MACE, emigration from Sweden or death, or until December 31, 2014.

### Biochemical analyses

Standard methods at the Department of Clinical Chemistry, Skåne University Hospital, Malmö, were used to analyze plasma lipids and creatinine. The Modification of Diet in Renal Disease formula (MDRD) was used to calculate the estimated glomerular filtration rate (eGFR)^17^.

A plasma aliquot was sent to the Science of Life Laboratory, Uppsala, Sweden for analysis of plasma sRAGE, performed by the Proximity Extension Assay technique^18^. Oligonucleotide-labelled antibody pairs were allowed to bind to sRAGE in the plasma samples. A PCR template was formed by adding a DNA polymerase that induced extension and joining of the two oligonucleotides, and they were further pre-amplified with universal primers. A specific primer was used to detect and quantify the individual DNA sequence that was created for sRAGE. The reactions were run on a microfluidic real-time quantitative PCR chip (96.96, Dynamic Array IFC, Fluidigm, Biomark) and read on a Biomark HD instrument. The between-run coefficient of variation for sRAGE was 11% and the within-run coefficient of variation was 9%, which is comparable with those obtained with commercially available ELISA kits. Data analysis was performed by a pre-processing normalization procedure using Olink Wizard for GenEx (Multid Analyses, Sweden). All data for sRAGE are presented in arbitrary units (au).

### Genotyping and Genome-wide association (pQTL) analysis

Genotyping was performed using the Illumina GWAS Chip (GSA v1 array) in the entire MDC cohort. All procedures followed the standard protocol. Certain single nucleotide polymorphisms (SNPs) were excluded due to genome mismatch, incorrect assignment of allelic variants in the array design, minor allele frequency (MAF) < 0.01, failed Hardy–Weinberg Equilibrium test at P < 1 × 10^−6^, call rate < 95% or failed genotype calling. Samples were excluded if they showed evidence of sex mismatch or had an overall sample call rate < 95%. The dataset was Imputed using the Michigan Imputation Server with reference panel of Haplotype Reference Consortium (HRC r1.1). Kinship was estimated using KING (v2.228) and individuals were censored to ensure no pair had closer than third degree kinship (Fig. 2).

After quality control, a total number of 4338 unrelated European subjects and 7,314,486 autosomal biallelic markers (MAF > 0.01 and imputation INFO score > 0.8) remained in the MDC-CV sub-cohort. The associations of log^2^-transformed protein levels of sRAGE with imputed variants were tested using an additive genetic model (linear Wald test) adjusted for age, age^2^, sex and the first to third principal components of population structure. Results are presented as beta coefficients given as normalized protein expression per allele increment. A cis-pQTL variant was defined as a locus within 1 megabase-pair (Mb) of the protein-coding gene. A trans-pQTL variant was ≥ 1 Mb from the gene. The proportion of variance of the sRAGE concentration explained by a pQTL SNP is shown as R^2^ (Table 2).

**Table 2:** Genome-wide significant loci associated with sRAGE levels in plasma.

Genomic locus	Chr	BP	rsID	A1	A2	EAF	Beta*	SE	P	R^2**†**^	Nearest gene
1	6	31109567	rs116653040	G	C	0.075	− 0.244	0.041	2.4E − 09	0.008	CCHCR1
	6	32151443	rs2070600	T	C	0.051	− 0.392	0.049	9.4E − 16	0.015	AGER
	6	32155581	rs204993	G	A	0.257	− 0.176	0.025	1.1E − 12	0.012	PBX2
2	12	617308	rs7306778	T	C	0.169	− 0.187	0.029	9.0E − 11	0.010	B4GALNT3

*Beta coefficient given as normalized protein expression per allele increment, adjusted for age, age^2^, sex and the first to third principal components of population structure.

^**†**^R^2^, proportion of variance explained.

Chr, chromosome; BP, base pair; A1, effect allele; A2, other allele; EAF, effect allele frequency; SE, standard error.

### Statistical analyses

A genome-wide significant P-value < 5 × 10^−8^ was used to select SNPs with a significant association with sRAGE. All SNPs with a squared Pearson correlation coefficient (r^2^) > 0.6 were considered to be in linkage disequilibrium (LD), and the SNPs with the strongest association (i.e. lowest P-value) to sRAGE within each LD block were used for further analyses. The median baseline sRAGE concentrations in individuals within the different allele groups of the selected SNPs were compared in a Kruskal Wallis analysis (Supplementary Table 1).

The associations between the SNPs and carotid IMT were explored in multivariate linear regression analyses with 2 different models of adjustment for potential confounders. Model 1 was adjusted for age and sex and Model 2 additionally included body mass index (BMI), current smoking, diabetes mellitus, triglycerides (TG), high density lipoprotein (HDL), low density lipoprotein (LDL), eGFR, systolic blood pressure, blood pressure medication, and statin medication. Skewed variables were logarithmically transformed before analysis. We analyzed the associations between the SNPs and carotid artery IMT at baseline, at reexamination, absolute IMT progression from baseline to reexamination, and the average IMT increase per year during follow-up. The normalized Z-scores of the IMT measurements were entered in the model as dependent variables and all the other factors, including the SNPs, were entered as independent variables. Consequently, the β-coefficients in Table 3 are expressed per 1-SD increase in the IMT variables, in order to allow comparisons between the effect size of the different SNPs on IMT at baseline, during follow-up and on delta IMT.

**Table 3:** Associations between sRAGE genetic variants and carotid IMT.

IMT	rs2070600		rs204993		rs116653040		rs7306778	
	ß (CI)	P	ß (CI)	P	ß (CI)	P	ß (CI)	P
Baseline								
1	− 0.01 (− 0.10–0.79)	0.828	0.02 (− 0.02–0.07)	0.350	0.001 (− 0.07–0.07)	0.980	0.01 (− 0.04–0.06)	0.675
2	− 0.02 (− 0.11–0.07)	0.689	0.03 (− 0.02–0.07)	0.213	− 0.02 (− 0.09–0.06)	0.654	− 0.01 (− 0.05–0.06)	0.827
Follow-up^**†**^								
1	0.06 (− 0.05–0.18)	0.298	0.01 (− 0.06–0.06)	0.875	− 0.03 (− 0.12–0.07)	0.581	0.04 (− 0.03–0.10)	0.310
2	0.06 (− 0.05–0.18)	0.275	0.01(− 0.06–0.06)	0.978	− 0.03 (− 0.13–0.06)	0.497	0.03 (− 0.04–0.09)	0.424
Absolute IMT Increase^‡^								
1	0.05 (− 0.07–0.17)	0.451	− 0.03 (− 0.09–0.03)	0.361	− 0.03 (− 0.13–0.07)	0.504	0.01 (− 0.06–0.08)	0.703
2	0.05 (− 0.07–0.17)	0.409	− 0.03 (− 0.09–0.03)	0.367	− 0.03 (− 0.13–0.07)	0.511	0.01 (− 0.06–0.08)	0.745
Yearly IMT increase								
1	0.04 (− 0.08–0.16)	0.528	− 0.03 (− 0.09–0.03)	0.333	− 0.03 (− 0.13–0.07)	0.536	0.006 (− 0.06–0.08)	0.876
2	0.04 (− 0.08–0.16)	0.494	− 0.03 (− 0.09–0.03)	0.346	− 0.03 (− 0.13–0.07)	0.509	0.003 (− 0.07–0.07)	0.940

Multivariate linear regression calculated per 1-SD IMT increase.

Adjustment models:

Model 1: age and sex.

Model 2: age, sex, BMI, current smoking, diabetes, LDL, HDL, TG, eGFR, systolic blood pressure, blood pressure lowering medication and statin medication.

^**†**^reexamination 2007–2012.

^‡^from baseline 1991–1994 to reexamination 2007–2012.

IMT, mean intima-media-thickness (mm) in the common carotid artery; BMI, body mass index; LDL, low density lipoprotein; HDL, high density lipoprotein; TG, triglycerides; eGFR, estimated glomerular filtration rate.

To examine the differences in baseline characteristics between event-free individuals and the subjects that suffered an incident event during follow-up (MACE or mortality), we used the Mann Whitney test for continuous variables and the chi-square test for dichotomous variables. Subjects having suffered a prevalent MACE before the baseline examination were excluded. The prospective associations between the identified SNPs and time from study inclusion to incident first-time MACE were examined in Cox regression analyses adjusted for age and sex (Model 1); and age, sex, BMI, current smoking, diabetes, ApoB, ApoA1, eGFR, systolic blood pressure, blood pressure medication, and statin medication (Model 2). The associations between the SNPs and mortality were explored in similarly adjusted Cox proportional hazard analyses. The median follow-up time to event was 21.2 (16.0–23.1) years for MACE (time to first MACE or death or end of follow-up) and 21.6 (18.3–23.3) years for mortality (time to death or end of follow-up).

A P-value of < 0.05 was considered to be statistically significant. The statistical analyses were performed with the SPSS statistics package 28 (IBM).

## Results

### Selection of sRAGE-associated SNPs in the MDC-CV cohort

The baseline characteristics of the 4338 unrelated European study participants with valid sRAGE and SNP measurements in the MDC-CV cohort are presented in Table 1. The median age at inclusion was 58 years and 40% were males. The median BMI was 25 kg/m^2^, approximately 20% of the study population were smokers, 16% were on blood pressure lowering medication, only 1.6% received statin medication and the prevalence of diabetes mellitus was 7.5%.

**Figure 3:**
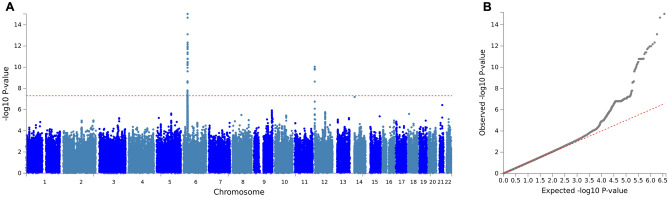
Plots of the genome-wide associations of plasma sRAGE in the MDC-CV cohort. (**A**) Manhattan plot, the dashed red line identifies the *P* = 5 × 10^−8^ level. *P* was − log10 transformed before plotting. (**B**) Quantile–quantile (Q–Q) plot with a genomic inflation factor (lambda) of 1.008.

**Figure 4:**
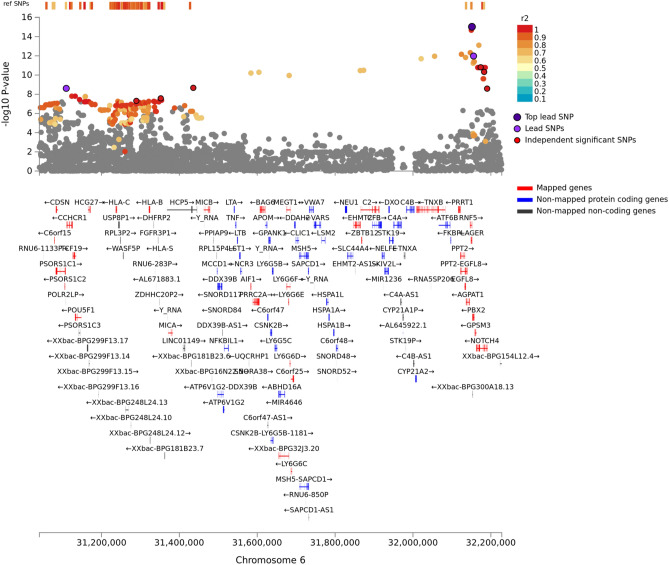
Regional plot of the chromosome 6 locus containing the lead sRAGE SNPs.

**Figure 5:**
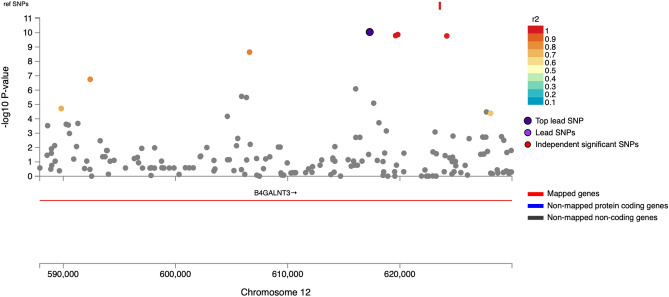
Regional plot of the chromosome 12 locus containing the rs7306778 SNP.

The GWAS analysis identified 48 genome-wide significant pQTL SNPs (*P* < 5 × 10^−8^), represented by four independent lead SNPs (r^2^ < 0.1), located in two genomic risk loci on chromosomes 6 and 12 (Figs. 3, 4, 5, Table 2). One of these mapped to the gene AGER that encodes RAGE (Fig. 4), containing the lead cis-pQTL SNP rs2070600 (minor allele T, MAF = 0.051) that explains 1.5% of plasma sRAGE variance. We also found a single trans-pQTL SNP, rs7306778, mapped to the gene *B4GALNT3* on chromosome 12 (Table 2, Fig. 5), explaining 1% of the sRAGE variance.

Functional annotation was performed on all SNPs in the loci that were in LD (r^2^ ≥ 0.6) with one of the independent significant pQTL SNPs using FUMA. In total, 350 out of the 544 annotated SNPs (64.3%) were in open chromatin regions. The combined annotation-dependent depletion (CADD) score was also examined to evaluate the deleteriousness of variants. We found one exonic non-synonymous pQTL SNP (rs2070600 in AGER, P = 9.5 × 10^−16^) with a high CADD score (24.5), suggesting a strong effect on sRAGE protein function.

The minor allele of all four selected SNPs was associated with significantly lower sRAGE levels (Supplementary Table 1). The largest decrease in circulating sRAGE was found for rs2070600, with an approximately 10% lowering of median sRAGE per minor allele.

Since a weak LD between rs2070600 and rs204993 was observed (LD r^2^ = 0.17), we performed a multivariable regression analysis to examine the potential combination effect of both SNPs on the circulating levels of sRAGE. The interaction term was not significant, suggesting the lack of genetic interaction between the two SNPs.

### Associations between sRAGE-associated SNPs and carotid IMT

In the MDC-CV cohort, we analyzed the associations between IMT at baseline, at follow-up and IMT progression from baseline to follow-up and the four lead SNPs. We found no significant associations between the SNPs and any of the IMT measurements, entered as dependent variables in multivariate linear regression models adjusted for age and sex (Model 1) or age, sex, cardiovascular (CV) risk factors and medication (Model 2) (Table 3).

### Prospective associations between sRAGE genetic variants, incident first-time MACE and mortality in the MDC cohort

The baseline characteristics of the 24,640 unrelated individuals of European ancestry included in the prospective study (Supplementary Table 2) were similar to those of the MDC-CV (Table 1), but with a slightly larger proportion of smokers (28% vs 20%), higher prevalence of statin medication (3% vs 1.6%) and lower prevalence of diabetes mellitus (4.5% vs 7.5%). There was a larger proportion of men among the individuals that suffered MACE during follow-up compared to MACE-free individuals, and these participants were significantly older, had a higher prevalence of hypertension, dyslipidemia, overweight, diabetes and smoking. The use of blood pressure- and lipid lowering medication was also higher (Supplementary Table 2). Similar differences in baseline characteristics were found between the subjects that died during follow-up and those who survived (Supplementary Table 3).

Incident first-time MACE after study inclusion occurred in 3469 (14%) participants without a history of prevalent coronary heart disease, and 8719 (35%) individuals died during follow-up (Fig. 2). In a Cox proportional hazards regression analysis adjusted for age and sex, we found a positive association between the minor allele (T vs C) of rs2070600 and incident first-time MACE [HR 1.13 95% CI (1.02–1.25), *P* = 0.016] (Table 4). The association remained significant after additional adjustment for cardiovascular risk factors and baseline medication (Table 4). The detailed results of the Cox proportional hazards regression analysis of the association between rs2070600 and incident first-time MACE are detailed in Supplementary Tables 4 and 5. We found no associations between the other SNPs and MACE, and none of the considered SNPs was related to mortality (Table 4).

**Table 4:** Associations between sRAGE genetic determinants and clinical outcomes.

SNPs	Incident first-time MACE^**†**^		Mortality	
	HR^#^ (CI)	P	HR^#^ (CI)	P
rs2070600				
1	1.13 (1.02–1.25)	0.016	1.03 (0.97–1.10)	0.338
2	1.11 (1.00–1.24)	0.045	1.03 (0.96–1.10)	0.475
rs204993				
1	0.96 (0.91–1.01)	0.134	1.01 (0.98–1.05)	0.439
2	0.98 (0.92–1.03)	0.418	1.02 (0.99–1.06)	0.242
rs116653040				
1	1.05 (0.96–1.14)	0.296	0.98 (0.92–1.03)	0.391
2	1.02 (0.93–1.12)	0.680	0.98 (0.92–1.04)	0.436
rs7306778				
1	0.98 (0.92–1.04)	0.526	0.99 (0.95–1.03)	0.601
2	0.98 (0.92–1.05)	0.602	1.00 (0.96–1.04)	0.990

^**†**^Incident first-time MACE from study inclusion to the end of follow-up. Subjects with prevalent MACE have been excluded from the analysis.

^#^Cox proportional hazard analysis. Time to event measured from study inclusion.

Adjustment models:

Model 1: age and sex.

Model 2: age, sex, BMI, current smoking, diabetes mellitus, ApoA1, ApoB, systolic blood pressure, blood pressure lowering medication and lipid lowering medication.

MACE, major adverse coronary event; BMI, body mass index; ApoA1, apolipoprotein A1; ApoB, apolipoprotein B.

## Discussion

We have previously shown that lower levels of plasma sRAGE are associated with increased risk for incident first-time MACE in the MDC-CV cohort^10^, which is also supported by the findings of other studies^19–21^. Here, we investigated whether the link between sRAGE and incident MACE is genetically determined. Our GWAS analysis on material from the MDC-CV cohort identified variations in the SNPs rs2070600, rs204993, rs116653040 and rs7306778 to be independent determinants of sRAGE levels at baseline. In the MDC cohort, we found that the minor T-allele of rs2070600 was associated with a 13% higher relative risk for first-time MACE during follow-up. The association remained significant after adjustment for traditional CV risk factors and baseline medication. To our knowledge, this is the first study to establish a direct independent link between a genetic RAGE determinant and CAD risk in a European population.

Activation of cell-bound RAGE leads to production of pro-inflammatory mediators, cell migration and tissue infiltration^3^, promoting inflammation and atherogenesis^22,23^. Conversely, RAGE deletion in ApoE^−/−^ mice reduces the size of atherosclerotic lesions^22,24^. The majority of sRAGE is generated by cleavage from the cell surface by MMP-9 (Matrix Metalloproteinase-9)^25^ and ADAM10 (a disintegrin and metalloproteinases domain-containing protein 10)^5^. This process is triggered by ligand binding to RAGE on the cell surface^5^. Only a small proportion of the total amount of circulating sRAGE is formed by alternate splicing and secretion^5^. sRAGE has been shown to have direct anti-inflammatory and atheroprotective properties in animal studies^26^. sRAGE treatment reduced atherosclerosis in diabetic mice independent of hyperglycemia and lipids^27,28^ and reduced vascular medial calcification in mice with chronic kidney disease^29^. Administration of sRAGE in diabetic rats rapidly reversed the vascular hyperpermeability and the endothelial dysfunction^30^ and it decreased neointimal expansion after vascular injury in both diabetic and non-diabetic rats^31^. Thus, it was reasonable to hypothesize that the increased CV risk linked to lower sRAGE levels is due to the partial loss of this protective mechanism. However, the minor alleles of rs204993, rs116653040 and rs7306778, associated with a similar decrease in plasma sRAGE as the minor allele of rs2070600, were not linked to MACE in our study. While rs204993, rs116653040 and rs7306778 are located in the vicinity of other genes (Table 2), rs2070600 is a known mutation in the AGER gene leading to changes in RAGE function*.* These results suggest that the links between rs2070600 and MACE are probably due to functional changes of the receptor, rather than to the sRAGE-lowering effect in itself.

The minor allele of rs2070600 is a missense mutation located in the exon 3 of AGER^32^^.^ The T-allele of rs2070600 induces a shift from Glycine to Serine in the amino acid position 82, leading to alterations in the structure and function of the ligand-binding V-domain of RAGE^32–34^. Similar to our findings, the presence of the T-allele of rs2070600 has previously been found to be associated with lower sRAGE levels in both Dutch and Korean populations, and has been related to a pro-inflammatory state characterized by higher levels of TNFα and hsCRP in plasma^35,36^. How the Gly82Ser polymorphism leads to reduced sRAGE in the circulation remains to be determined. A previous study of the functional importance of the Gly82Ser polymorphism in humans has shown that mononuclear phagocytes isolated from homozygous 82G/82G or heterozygous 82G/82S subjects express similar levels of RAGE. However, RAGE ligand challenge induced a 4.5-fold increase in phosphorylated MEK1/2 in 82G/82S phagocytes, compared to only 2.6-fold in 82G/82G phagocytes, ultimately leading to enhanced production of pro-inflammatory cytokines and MMPs^37^. Thus, the presence of the minor rs2070600 allele enhances the propensity of RAGE activation. In turn, RAGE activation leads to receptor internalization^26,38^ and reduced availability for cleavage, which might explain the lower sRAGE levels in T-allele carriers. Theoretically, the reduction of sRAGE levels in the circulation may also be due to increased resistance of RAGE to cleavage. However, considering that the Gly82Ser mutation occurs in the ligand-binding V-domain situated far away from the single transmembrane helix anchor of the receptor, this hypothesis is less plausible.

We are the first to show an independent link between rs2070600 and incident first-time MACE in a single-site large European cohort. The only other study to date to detect a significant link between the RAGE Gly82Ser polymorphism and CAD is a meta-analysis of seven studies including a total of 3033 cases and 2732 controls of Chinese ancestry^39^. Our data confirm these findings and demonstrate that the relationship between this RAGE-modifying SNP and CVD is present in both Chinese and Europeans. In an earlier study on 9017 whites and 2871 blacks from the Atherosclerosis Risk in Communities (ARIC) study, Maruthur et al*.* also identified rs2070600 as the strongest determinant of lower sRAGE in the white population^40^. The association between rs2070600 and sRAGE was not present in blacks. The HR for CAD risk per minor rs2070600 allele was equal to the one found in our study [HR 1.12 (95% CI 0.91–1.38)], but the association was not significant. The associations between rs2070600 polymorphisms and CVD have also been explored in three other meta-analyses^41–43^. The first included 10 studies exploring the associations between rs2070600 and CVD, defined as coronary stenosis or myocardial infarction, with a total subject size of 2741 patients and 4336 controls^42^. The second meta-analysis combined data from 14 studies, totaling 2145 CAD patients and 4966 controls^41^. However, only two of the included cohorts had over 300 subjects, and the largest cohort considered included 1632 subjects from the Framingham offspring study^33^. The third meta-analysis included 4402 cases and 6081 controls^43^. Interestingly, all these studies found an overall HR of 1.12 per serine-encoding allele of rs2070600 to develop CAD, but neither reached statistical significance. The main difference between these previous studies and ours is population size. The MDC cohort is almost three times larger than ARIC, the nearest cohort in term of size, and is equal to all these previous studies combined. The effect allele frequency (EAF) of rs2070600 is only 5.1%, and the SNP is only responsible for a small percentage of sRAGE variation in the population. Thus, it is likely that these previous studies have been underpowered.

Our study has some important limitations. Firstly, our analysis is restricted to a European population and our findings may therefore not be generalizable to other populations. Secondly, carotid IMT data is only available in the MDC-CV cohort, but not in the large MDC population. Thus, the analyses of the associations between rs207600 and IMT might have been underpowered. Thirdly, as we have used the PLA method to measure sRAGE, plasma concentrations are expressed in relative units and cannot be directly compared with the published values in studies that have used other methods. However, as our GWAS analysis has identified the same SNP to be the main determinant of sRAGE as in previous studies, the final results of the studies are comparable. Lastly, since rs2070600 was identified by exploring genetic variants affecting plasma sRAGE, other mutations affecting RAGE function but with limited impact on sRAGE levels could not be detected by our method.

In conclusion, we show for the first time that European carriers of the minor T-allele of the AGER SNP rs2070600 have an increased risk to suffer MACE, independently of CV risk factors. Our initial GWAS analysis identified four genetic variants as independent determinants of low plasma sRAGE, but only rs2070600 was related to MACE. The Gly82Ser polymorphism induced by the T allele of rs2070600 renders the receptor more immunologically active. It is likely that the relationship of rs2070600 with MACE is driven by the functional modification of RAGE rather than by the sRAGE-lowering effect. These findings provide genetic support for a causal role of the RAGE receptor in CVD.

The lack of association between the other sRAGE-lowering SNPs and MACE does not contradict previous studies showing negative relationships between plasma sRAGE and CV risk. rs2070600, the strongest genetic sRAGE determinant in our study and in the work of others^40^, is only responsible for 1.5% of plasma sRAGE variation, so the majority of this variation is not genetically determined. Thus, we cannot exclude that sRAGE lowering by other factors contribute to increased CV risk by reducing the capacity of sRAGE to bind and inactivate RAGE ligands.

### Supplementary Information

Supplementary Tables.

## Data Availability

The data that support the findings of this study are available from the corresponding author upon request.
