# Supplementary material for: The Gly82Ser polymorphism in the receptor for advanced glycation endproducts increases the risk for coronary events in the general population
Source: Sci Rep. 2024 May 21;14:11567. doi: 10.1038/s41598-024-62385-5 (PMC11109115; doi:10.1038/s41598-024-62385-5)
Supplement: Supplementary file 1 — Supplementary Tables. [file 41598_2024_62385_MOESM1_ESM.pdf]

The Gly82Ser polymorphism in the receptor for advanced glycation end products increases the risk for coronary events in the general population

Helena Grauen Larsen[1,2], Jiangming Sun[1], Marketa Sjögren[1], Yan Borné[1], Gunnar Engström[1], Peter Nilsson[1], Marju Orho-Melander[1], Isabel Goncalves[1,2], Jan Nilsson[1], Olle Melander[1,3], Alexandru Schiopu[3,4,5,*]

[1] Lund University, Department of Clinical Sciences Malmö, Lund, 22100, Sweden
[2] Skane University Hospital, Department of Cardiology, Lund, 22241, Sweden
[3] Skane University Hospital, Department of Internal Medicine, Lund, 22241, Sweden
[4] Lund University, Department of Translational Medicine, Lund, 22100, Sweden
[5] Nicolae Simionescu Institute of Cellular Biology and Pathology, Bucharest, 050568, Romania

Supplementary table 1. sRAGE levels according to allele frequency for the four main sRAGE-determinant SNPs identified by GWAS.

	sRAGE (au)				
**SNPs**	**Homozygote major allele**	**Heterozygote**	**Homozygote minor allele**	**P***	**N (maj/het/min)**
rs2070600	20.1 (16.4-24.2)	17.9 (14.3-21.6)	15.1 (11.9-15.1)	<0.001	3909/419/10
rs204993	20.6 (16.7-24.7)	19.1 (15.6-23.0)	18.9 (15.3-22.7)	<0.001	2371/1702/265
rs116653040	20.1 (16.4-24.3)	18.7 (15.0-22.8)	17.7 (14.8-22.6)	<0.001	3697/618/23
rs7306778	20.3 (16.5-24.5)	19.1 (15.3-23.4)	18.2 (14.9-22.4)	<0.001	3012/1203/123

sRAGE values expressed as median (IQR) arbitrary units (au).

*Kruskal Wallis Test

Maj, major allele homozygote; het, heterozygote; min, minor allele homozygotes.

Supplementary table 2. Baseline characteristics in subjects with incident first-time MACE compared to MACE-free subjects.

Characteristics	**Whole cohort*** N=24640	**Incident first-time MACE** N=3469	**No MACE** N=20550*	***P***[#]
Age (years)	58 (51-64)	61 (55-66)	57 (51-63)	<0.001
Male gender, n (%)	9850 (40.0)	2080 (60.0)	7287 (35.5)	<0.001
BMI (kg/m^2)	25.3 (23.0-28.0)	26.3 (23.9-28.9)	25.1 (22.9-27.8)	<0.001
Diabetes, n (%)	1113 (4.5)	310 (8.9)	711 (3.5)	<0.001
Current smoking, n (%)	6499 (28.1)	1069 (30.8)	5305 (25.8)	<0.001
Medication use				
Lipid lowering medication, n (%)	718 (2.9)	155 (4.5)	338 (1.6)	<0.001
Blood pressure medication, n (%)	4284 (17.4)	881 (25.4)	2989 (14.5)	<0.001
Lipids				
ApoA1 (mg/dL)	154 (137-174)	146 (130-164)	156 (139-176)	<0.001
ApoB (mg/dL)	105 (89-123)	115 (99-133)	103 (87-121)	<0.001
Blood pressure				
Systolic (mmHg)	140 (126-154)	148 (134-160)	138 (125-150)	<0.001
Diastolic (mmHg)	85 (80-90)	88 (80-95)	85 (80-90)	<0.001

The data are presented as median (interquartile range) for the continuous variables.

[*] Individuals with prevalent MACE prior to enrolment (N=618) have been excluded from the subsequent analysis. The total number of subjects included in the analysis was 24,019.

[#] Comparing individuals with and without incident first-time MACE, Chi square test on dichotomous variables and Mann Whitney test on continuous variables.

MACE, major adverse coronary event; BMI, body mass index; LDL, low density lipoprotein; HDL, high density lipoprotein; TG, triglyceride.

Supplementary table 3. Baseline characteristics depending on mortality status in the MDC cohort.

Characteristics	Whole cohort<br>N=24640	Dead<br>N=8719	Alive<br>N=15680	*P**
Age, (years)	58 (51-64)	64 (58-68)	54 (50-61)	<0.001
Male gender, n (%)	9850 (40.0)	4450 (51.0)	5306 (33.8)	<0.001
BMI, (kg/m^2)	25.3 (23.0-28.0)	25.8 (23.4-28.7)	25.0 (22.9-27.7)	<0.001
Diabetes, n (%)	1113 (4.5)	676 (7.8)	431 (2.7)	<0.001
Current smoking, n (%)	6499 (28.1)	2713 (31.1)	3727 (23.8)	<0.001
Medication use				
Lipid lowering medication, n (%)	718 (2.9)	389 (4.5)	326 (2.1)	<0.001
Blood pressure medication, n (%)	4284 (17.4)	2236 (25.6)	2022 (12.9)	<0.001
Lipids				
ApoA1 (mg/dL)	154 (137-174)	151 (134-171)	156 (139-176)	<0.001
ApoB (mg/dL)	105 (89-123)	109 (94-127)	103 (87-120)	<0.001
Blood pressure				
Systolic (mmHg)	140 (126-154)	148 (132-160)	136 (122-150)	<0.001
Diastolic (mmHg)	85 (80-90)	88 (80-95)	84 (78-90)	<0.001

The data are presented as median (IQR) for the continuous variables. Data on the vital status at the end of the study was missing in 238 subjects that were excluded from the analysis.

*Comparing individuals that died with survivors, Chi square test on dichotomous variables and Mann Whitney test on continuous variables.

BMI, body mass index; LDL, low density lipoprotein; HDL, high density lipoprotein; TG, triglyceride.

Supplementary table 4. Detailed Cox regression analysis of the association between the minor allele of rs2070600 and incident MACE, adjusted for age and sex

**Variables**	**Unstandardized β coefficient**	**Standard error for β**	**Wald**	**HR (CI)**	**p**
rs2070600	0.12	0.05	5.83	1.13 (1.02 - 1.25)	0.016
Age	0.07	0.002	795.32	1.07 (1.07 - 1.08)	<0.001
Sex (female)	-0.95	0.04	749.43	0.39 (0.36 - 0.41)	<0.001

Supplementary table 5. Detailed Cox regression analysis of the association between the minor allele of rs2070600 and incident MACE, adjusted for age, sex and CV risk factors

**Variables**	**Unstandardized β coefficient**	**Standard error for β**	**Wald**	**HR (CI)**	**p**
rs2070600	0.11	0.05	4.00	1.11 (1.00 - 1.24)	0.045
Age	0.06	0.003	406.02	1.06 (1.05 - 1.06)	<0.001
Sex (female)	-0.76	0.04	376.66	0.47 (0.44 - 0.51)	<0.001
BMI	0.02	0.005	10.97	1.016 (1.01 - 1.03)	<0.001
Smoking	0.52	0.04	170.71	1.68 (1.56 - 1.82)	<0.001
Diabetes	0.62	0.06	89.36	1.86 (1.64 - 2.12)	<0.001
ApoA1	-0.01	0.001	103.76	0.99 (0.99 - 0.99)	<0.001
ApoB	0.01	0.001	190.07	1.01 (1.01 - 1.01)	<0.001
Systolic blood pressure	0.01	0.001	128.66	1.01 (1.01 - 1.01)	<0.001
Blood pressure medication	0.30	0.04	46.94	1.35 (1.24 - 1.47)	<0.001
Lipid lowering medication	0.54	0.08	40.83	1.72 (1.45 - 2.02)	<0.001

BMI, body mass index; ApoA1, apolipoprotein A1; ApoB, apolipoprotein B.
